# Psychological factors and the uptake of preventative measures in *BRCA1/2* pathogenic variant carriers: results of a prospective cohort study

**DOI:** 10.1186/s13053-022-00244-y

**Published:** 2022-12-19

**Authors:** Julia Dick, Anja Tüchler, Anne Brédart, Frank Vitinius, Kirsten Wassermann, Kerstin Rhiem, Rita K. Schmutzler

**Affiliations:** 1 Center for Hereditary Breast and Ovarian Cancer, Faculty of Medicine and University Hospital Cologne, University of Cologne, Kerpener Str. 62, 50937 Cologne, Germany; 2 Institut Curie, Supportive Care Department, Psycho-oncology Unit, 26 rue d’Ulm, 75005 Paris Cedex 05, Paris, France; 3 Paris University, Psychology Institute, Psychopathology and health process laboratory UR4057, 71 avenue Edouard Vaillant, 92774 Boulogne-Billancourt, France; 4 Department of Psychosomatics and Psychotherapy, Faculty of Medicine and University Hospital Cologne, University of Cologne, Cologne, Germany; 5 Psychologische Praxis, Bonn, Germany

**Keywords:** Risk reduction behavior, Breast cancer, familial, Anxiety, Decision making, Risk-reducing mastectomy

## Abstract

**Background:**

Women carrying *BRCA1/2* pathogenic variants are exposed to elevated risks of developing breast cancer (BC) and are faced by a complex decision-making process on preventative measures, i.e., risk-reducing mastectomy (RRM), and intensified breast surveillance (IBS). In this prospective cohort study we investigated the effect of anxiety, personality factors and coping styles on the decision-making process on risk management options in women with pathogenic variants in *BRCA1/2*.

**Methods:**

Breast cancer unaffected and affected women with a pathogenic variant in the *BRCA1* or *BRCA2* gene were psychologically evaluated immediately before (T0), 6 to 8 weeks (T1) and 6 to 8 months (T2) after the disclosure of their genetic test results. Uptake of RRM and IBS was assessed at T2. Psychological data were gathered using questionnaires on risk perception, personality factors, coping styles, decisional conflict, depression and anxiety, including the Hospital Anxiety and Depression Scale (HADS). We performed tests on statistical significance and fitted a logistic regression based on significance level.

**Results:**

A total of 98 women were included in the analysis. Baseline anxiety levels in women opting for RRM were high but decreased over time, while they increased in women opting for intensified breast surveillance (IBS). Elevated levels of anxiety after genetic test result disclosure (T1) were associated with the decision to undergo RRM (*p* < 0.01; OR = 1.2, 95% CI = 1.05–1.42), while personal BC history and personality factors seemed to be less relevant.

**Conclusions:**

Considering psychosocial factors influencing the decision-making process of women with pathogenic variants in *BRCA1/2* may help improving their genetic and psychological counselling. When opting for IBS they may profit from additional medical and psychological counselling.

**Trial registration:**

Retrospectively registered at the German Clinical Trials Register under DRKS00027566 on January 13, 2022.

## Background

Women carrying pathogenic germline variants (PVs) in the *BRCA1/2* genes face elevated risks of developing breast cancer (BC) and ovarian cancer (OC). *BRCA1* pathogenic variants expose women to a lifetime risk of 72% (95% confidence interval [CI] 65–79%) for BC, while *BRCA2* pathogenic variant carriers face a risk of 69% (95% CI 61–77%) [1]. The increase in contralateral BC risk depends on the age at first diagnosis and the affected gene [2–4].

These elevated cancer risks confront women with different options of preventative measures for BC, namely intensified breast surveillance (IBS) and risk-reducing mastectomy (RRM). The purpose of IBS is the early detection of newly developing breast carcinomas [5, 6]. It has been shown to reduce mortality rates [7], but cannot reduce the risk of developing BC. Bilateral (BRRM) and contralateral risk-reducing mastectomy (CRRM) can significantly reduce BC risk [8, 9] and mortality [10, 11] in women with *BRCA1/2* pathogenic variants. RRM has also been shown to reduce anxiety levels in women at risk for developing BC: McCarthy, Hamill [12] found that general anxiety was significantly reduced 1 year after BRRM including reconstruction and they remained decreased at 2 years follow-up. However, RRM is known to produce adverse consequences in the wellbeing of women [13], especially regarding breast related body image [14, 15] as well as sensuality and sexuality [16, 17]. Studies have reported on feelings of dissatisfaction with the results of RRM surgery [17], making a small percentage of women even regret their decision [18–22].

In light of the surgical procedure’s irreversible nature, women with PVs in *BRCA1/2* are confronted with complex genetic and statistical information on their approximate age-related disease probabilities associated with their genetic predisposition. Moreover, they face difficult tradeoffs when deciding on risk management options [23], including the side effects of preventative and surgical measures, and anticipated long-term results. Personal values, expectations, anticipations, and beliefs have been shown to influence decision making on risk management options, e.g., the decision on prophylactic measures and/or intensified breast surveillance [24–26]. Understanding the psychological factors that may influence the decision-making process is paramount for patient-centered genetic counselling [27]. For example, distress levels and risk perception have been shown to play an important role in women’s intentions to undergo risk-reducing surgery [28]. In addition, cancer worry, risk perception, anticipated regret in case of a future BC diagnosis and having children are sought to be associated with the intention to undergo prophylactic mastectomy in healthy women at high risk for BC [25, 29]. In a literature review, Glassey et al. [17] found contradictory evidence on the relation between anxiety levels and choice for preventative option. The importance of coping styles (monitoring and blunting) has been reported for information needs in oncological settings [30] as well as cancer worry [31]. The aim of this prospective observational study was to determine the influence of psychological and psychosocial factors and coping styles and personality treats on the decision to undergo either RRM or IBS after genetic test result disclosure.

## Methods

### Participants and inclusion criteria

Study participants were recruited prospectively at the Centre for Hereditary Breast and Ovarian Cancer at the University Hospital of Cologne between June 2013 and December 2014.

To be included in this study, women had to be 18 years or older. They needed to be able to give written consent to participate in the study and have an adequate understanding of German. We included women with a PV in the *BRCA1* and *BRCA2* gene, respectively. Both BC-unaffected women (in the following referred to as ‘healthy’) and women unilaterally affected by BC were considered. Women diagnosed with OC or bilateral BC were excluded from the study. This study was reviewed and approved by the Ethics Committee of the University Hospital of Cologne (Date of approval: 22.05.2013/No. 13–028). Written consent to study participation was obtained from all women.

### Clinical setting: genetic counseling and risk communication

All participants received pre- and post-test genetic counseling by specialists in medical genetics. Pre-test genetic counseling included general information on hereditary BC and OC, PV testing and possible preventative or therapeutic options including information on potential benefits and harms, risks, side effects, and long-term follow up. Post-test genetic counseling included information on individual test results, absolute risks of developing BC, risk-adjusted preventative options, and competing risks associated with unilateral BC or other diseases.

Individual statistical risks for developing BC were calculated using the Breast and Ovarian Analysis of Disease Incidence and Carrier Estimation Algorithm (BOADICEA) V3 model [32]. Information on BC and/or OC and *BRCA1/2* pathogenic variant status for first-degree and second-degree family members was included. We communicated both the 5-year risk for developing BC and OC and the risk of recurrence and metastases as well as the risk for developing contralateral BC for BC affected women.

### Measures

We collected data on age, marital status, parity, and number of children as well as socio-economic characteristics on educational level, occupation, and employment status. Clinical data and family history were retrieved from medical files.

Psychological evaluation comprised the following measures. An overview on questionnaires and measurement points is displayed in Table 1.

- The HADS-D [33], the German version of the internationally used Hospital Anxiety and Depression Scale (HADS) [34], for measuring emotional. It comprises 14 items, seven each relating to either generalized anxiety or depression. For both scales, a sum score of 0 to 7 for either subscale is considered inconspicuous (‘non cases’) by the authors [34]; scores between 8 and 10 are considered conspicuous, suggesting borderline or doubtful cases of anxiety or depression, while a score above 10 indicate a ‘case’ on either scale. In this analysis, we examine anxiety as a dichotomous variable with scores of 7 or lower as “inconspicuous levels of anxiety”, and “increased/clinically relevant anxiety” with scores of 8 and higher. This allows for the identification of all possible cases including subsyndromal manifestations of anxiety, but heightens the chances of false positives [34].
- The Freiburg Personality Inventory (FPI-R), a personality inventory used for assessing personality factors in clinical diagnostics in Germany [35]. It comprises 138 statements to which participants agree or disagree. The FPI-R aims at assessing 12 personality factors: life satisfaction, social orientation, achievement orientation, inhibition, irritability, aggression, stress, somatic complaints, health concerns, and openness, as well as two secondary factors: extraversion and emotionality/neuroticism. Higher scores indicate higher expression on each respective scale.
- The Decisional Conflict Scale (DCS), commonly used to identify a state of decisional conflict in difficult situations [36, 37]. Its total score ranges from 0 (no decisional conflict) to 100 (extremely high decisional conflict). The scale comprises five subscales (uncertainty, informed, values clarity, support, effective decision) with scores ranging from 0 to 100. Higher scores indicate more problems dealing with decisional conflict.
- The Frankfurt Monitoring and Blunting Scale (FMBS) [38], a revised version of the Miller Behavioral Style Scale (MBSS) [39], designed to classify coping styles, i.e. the preference for either seeking information (‘*monitoring’*) or avoiding it (‘*blunting’*) in controllable and uncontrollable situations, respectively. Eight behavioral choices are rated on a four-point rating scale ranging from 1 (“Completely disagree”) to 4 (“Completely agree”).
- Information on women’s decision on preventative measures (IBS or RRM) at T1 as well as actual uptake of preventative measures at T2 was collected.
- A visual analogue scale ranging between 1 and 100% measured 5-year BC risk perception.

**Table 1:** Overview on questionnaires used and data gathered at different measurement time points

	Time Point	Questionnaires	Specification
**T0**	Prior to genetic test result disclosure	▪ HADS	▪ Anxiety and depression
**T1**	6–8 weeks after test result disclosure	▪ HADS ▪ FPI-R ▪ FMBS ▪ DCS ▪ Intention on RRM/IBS ▪ Sociodemographic data ▪ Family history ▪ Clinical data/cancer history ▪ Risk perception	▪ Anxiety and depression ▪ Personality ▪ Coping styles ▪ Decisional conflict
**T2**	6–8 months after test result disclosure	▪ HADS ▪ Actual uptake of RRM/IBS	▪ Anxiety and depression

*Abbreviations*: *T* Measurement time points, *HADS* Hospital Anxiety and Depression Scale, *RRM* Risk reducing mastectomy, *IBS* Intensified breast surveillance, *DCS* Decisional Conflict scale, *FMBS* Frankfurt Monitoring and Blunting Scale, *FPI-R* Freiburg Personality Inventory

### Statistical analyses

We report on mean values (*M*) and standard deviation (*SD*) of two subgroups: women who opted for IBS (IBS group) or underwent RRM (RRM group) at T2 (6–8 months after genetic test result disclosure). We used Pearson’s *Χ*²-test for categorical variables and the *t*-test for continuous variables. The threshold for statistical significance was set at *p* < .05 and adjusted for effects of multiple testing. All *p*-values were two-sided. To inform on changes of anxiety over time, variance of analyses (ANOVA) with repeated measures was performed using general linear modeling. The Huynh-Feldt adjustment was used to correct for violations of sphericity.

The influence of psychosocial factors (independent variables) on the decision for either RRM or IBS (dependent variable) were determined by using binary logistic regression analyses using stepwise forward selection of variables based on the likelihood ratio test. Our final model was fitted with variables with a *p*-value < .05 (Cut-off). All statistical analyses were performed using SPSS 27.0.

## Results

### Basic characteristics

**Fig. 1:**
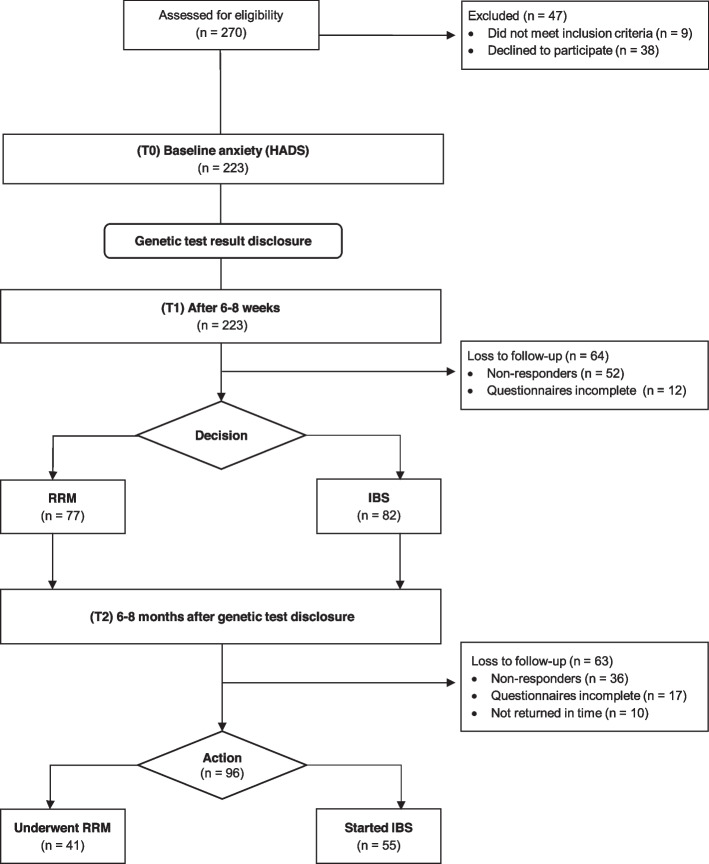
Study flow chart

We recruited 159 women with deleterious *BRCA1* and *BRCA2* pathogenic variants. An overview on study participation is presented in study flow chart (Fig. 1). A total of 98 (61.6% response rate) participants returned the questionnaires on all three measuring points and were included in the final analysis. Basic characteristics and socio-demographics are presented in Table 2. Participant’s ages ranged from 20 to 70 years (*M* = 42.2, *SD:* 10.9), 40 women were BC-unaffected, 58 women were diagnosed with invasive unilateral BC. Two thirds (62%) of all participants had children, 69% were married or in a relationship. Every fifth woman was under the age of 20 when a close female relative had died from BC. Patients were asked on current mental health conditions and/or psychiatric disorders via a self-administered questionnaire. Four women reported suffering from depression or depressive episodes, one woman reported having anxiety and panic attacks.

**Table 2:** Cohort sample characteristics of study participants that returned questionnaires at all three time points.

	Total		RRM group		IBS group		*p* -value
	N	%	n	%	n	%	
**Total**	97^a^	100	33	33.3	64	66.7	
**Age** (*n* = 97)							
≤ 29	14	14.4	5	35.7	9	64.3	.911
30–39	21	21.6	6	28.6	15	71.4	
40–49	40	41.2	15	37.5	25	62.5	
50–59	17	17.5	6	35.3	11	64.7	
≥ 60	5	5.2	1	20.0	4	80.0	
**Educational level** (*n* = 96)							
Low/intermediate	48	50.0	12	25.0	36	75.0	.083
High/university	48	50.0	20	41.7	28	58.3	
**Children** (*n* = 93)							
Yes	59	63.4	22	37.3	37	62.7	.287
No	34	36.6	9	26.5	25	73.5	
**Marital status** (*n* = 95)							
Married or in relationship	69	72.6	25	36.2	44	63.8	.392
Single	26	27.4	7	26.9	19	73.1	
**BC diagnosis** (*n* = 97)							
Yes	57	58.8	19	33.3	38	66.7	.865
No	40	41.2	14	35.0	26	65.0	
**Mother affected by or died from BC** (*n* = 97)							
Yes	56	57.7	21	37.5	35	62.5	.398
No	41	42.3	12	29.3	29	70.7	
**Sister affected by or died from BC** (*n* = 97)							
Yes	32	33.0	11	34.4	21	65.6	.959
No	65	67.0	22	33.8	43	66.2	
**Grandmother affected by or died from BC** (*n* = 97)							
Yes	27	27.8	8	29.6	19	70.4	.571
No	70	72.2	25	35.7	45	64.3	
**Aged < 20 when family member died from BC** (*n* = 97)							
Yes	22	22.7	6	27.3	16	72.7	.447
No	75	77.3	27	36.0	48	64.0	
**Statistical 5-year BC risk** (BOADICEA) at T1 (*n* = 92)							
Mean			10.0%		9.8%		.813
*SD*			4.0		4.5		
**Lifetime BC risk perception at T1** (*n* = 54)							
Mean			68.1%		55.3%		.180
*SD*			33.9		25.9		
**5-year BC risk perception T2** (*n* = 94)							
Mean			9.1%		28.2%		<.001
*SD*			12.8%		24.8%		

*Abbreviations*: *SD* Standard deviation, *RRM* Risk-reducing mastectomy, *IBS* Intensified breast surveillance, *BC* Breast cancer, *BOADICEA* Breast and Ovarian Analysis of Disease Incidence and Carrier Estimation Algorithm, *CI* Confidence interval

^a^Missing value: 1

### Non-responder analysis

Women lost to follow-up (*N* = 61) were compared to the women participating in the study until T2 (*N* = 98). No difference was found on BC status (i.e., women diagnosed with unilateral BC, and cancer unaffected women), educational status and age. However, non-responders (women not returning their questionnaires at all three measurement points) had significantly higher levels of anxiety compared to active study participants at T2 (*M* = 8.33 vs. 6.92, *p* < .05; data not displayed).

### Factors associated with the decision on preventative measures

#### Socio-demographic data, family history, BC status and risk

A total of 97 women provided information on choice of preventative measure at T2 (missing values: 1). No difference was found between women who underwent RRM (*N* = 33) and women who chose IBS (*N* = 64) at T2 in terms of socio-demographics, risk status, family characteristics and number of family members affected by or deceased from BC. We found no difference between participant’s BC status (healthy or unilaterally affected) and psychosocial characteristics as well as their respective choice of preventative option at T2: 19 BC-affected and 14 unaffected women underwent RRM (33.3% vs. 35.0%, *p* = .87).

Mean BOADICEA V3 5-year BC risk at genetic test result disclosure was 9.78% (*SD* 4.31) and did not differ significantly between groups (*M*_*RRM*_ = 9.97%, *SD* = 4.45% and *M*_*IBS*_ = 9.75%, *SD* = 4.03%; *p* = .81).

#### Anxiety

**Fig. 2:**
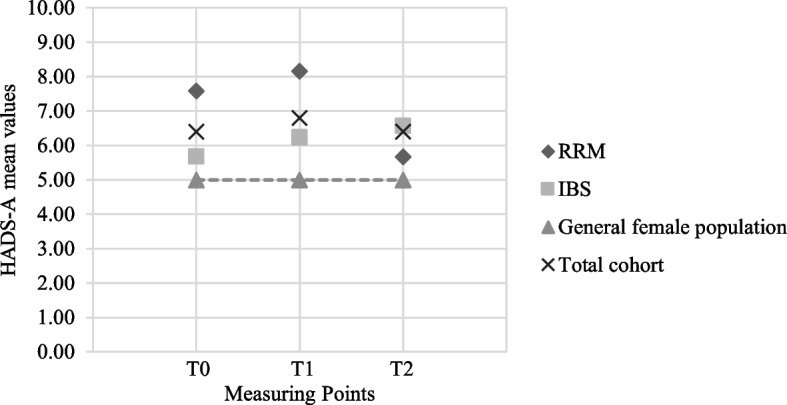
Anxiety levels over time. Anxiety level mean scores T0 through T2, classified by decision on preventative measure (T1). Abbreviations: T = measuring point, T0 = at baseline before disclosure of genetic test result, T1 = 6 to 8 weeks after the genetic test result disclosure, T2 = 6–8 months after the genetic test result disclosure, RRM = risk-reducing mastectomy (including bilateral and contralateral risk-reducing mastectomy), IBS = intensified breast surveillance

When compared to the population average for German females (*M* = 5.0, *SD* = 3.4) [40], our study group showed slightly elevated (though not clinically relevant) levels of anxiety at every measured time point (Table 3, Fig. 2). Overall, there was no statistically significant difference of anxiety mean levels between measurement points, with *F*(1.57, 140.82) = 1.22, *p* = .292 (Huynh-Feldt correction).

**Table 3:** HADS anxiety and depression levels over time. Scores < 8: inconspicuous/non-case; scores ≥8: borderline cases of anxiety/depression. T0: at baseline (before genetic test result disclosure), T1: 6–8 weeks after genetic test result disclosure, T2: 6–8 months after genetic test result disclosure

**T0**	**Total**		**RRM group**		**IBS group**		***p*** **-value**
	N	%	N	%	N	%	
	97	100	33	33.3%	64	66.7%	
Anxiety							
*M (SD)*	6.3 (4.1)		7.6 (4.6)		5.7 (3.7)		.03
< 8	56	57.7%	22	39.3%	34	60.7%	.201
≥ 8	41	42.3%	11	26.8%	30	73.2%	
Depression							
*M* (*SD*)	2.7 (2.7)		3.1 (2.5)		2.5 (2.8)		.32
**T1**	**Total**		**RRM group**		**IBS group**		***p*** **-value**
	N	%	N	%	N	%	
	96	100%	32	33.3%	64	66.7%	
Anxiety							
*M (SD)*	6.9 (4.1)		8.2 (4.4)		6.2 (3.9)		.03
< 8	60	62.5%	16	26.7%	44	73.3%	.075
≥ 8	36	37.5%	16	44.4%	20	55.6%	
Depression							
*M* (*SD*)	3.2 (3.2)		3.2 (3.0)		3.2 (3.3)		.95
**T2**	**Total**		**RRM group**		**IBS group**		***p*** **-value**
	N	%	N	%	N	%	
	97	100%	33	33.3%		66.7%	
Anxiety							
*M (SD)*	6.3 (3.8)		5.7 (3.9)		6.6 (3.8)		.27
< 8	56	57.7%	22	39.3%	34	60.7%	.201
≥ 8	41	42.3%	11	26.8%	30	73.2%	
Depression							
*M (SD)*	3.0 (3.0)		2.2 (3.0)		3.4 (3.2)		.05

*Abbreviations*: *M* Mean, *SD* Standard deviation, *RRM* Risk-reducing mastectomy, *IBS* Intensified breast surveillance

However, anxiety scores were significantly higher in the RRM group compared to the IBS group at baseline (T0: *M*_*RRM*_ = 7.6, *M*_IBS_ = 5.7, *p* = .03) and after genetic test result disclosure (T1: *M*_RRM_ = 8.2, *M*_IBS_ = 6.2, *p* = .03). It is of note, that 6 weeks after genetic test result disclosure, the anxiety mean value of women opting for RRM at T2 was > 8. More precisely, 16 of 32 (50.0%, one missing value) women had increased levels of anxiety (≥ 8), ten of which (10 of 32; 31.3%) had anxiety levels of > 10.

At T2, anxiety decreased in women who underwent RRM, while slightly increasing among women with IBS. In the IBS group, a total of thirty women displayed elevated levels of anxiety (46.9% of IBS), while eleven women in the RRM group had elevated levels of anxiety even after 6–8 months and RRM (33.3% of RRM group).

Overall, 16 of 36 women (44.4%) with increased anxiety scores at T1 underwent RRM at T2, whereas 55.6% (20 of 36) opted for IBS. Accordingly, 44 of 60 (73.3%) women with low anxiety scores opted for IBS, while 16 (26.7%) underwent RRM at T2. Irrespective of whether the women have acute BC or have ever had BC in their lives, 37 of 97 women (38.1%) showed increased anxiety scores of ≥8 at T1. Of these, 25 (67.6%) intended to undergo RRM at T1, 15 of which had undergone RRM at T2.

#### Depression

Mean level of HADS-Depression remained consistently low over time. Compared to the female German general population (*M* = 4.7, *SD* = 3.9) [40], scores were generally low. Mean level of depression did not differ between groups before (T0) and 6–8 weeks after genetic test result disclosure (T1). At T2, mean levels in the IBS group rose to 3.4 and differed significantly from the RRM group, while remaining at inconspicuous level overall.

#### BC risk perception

Lifetime BC risk perception at T1 did not differ significantly between IBS and RRM groups (55.3 and 68.1% respectively). At T2, 6–8 months after genetic test result disclosure, perceived 5-year BC risk differed significantly between women who underwent RRM and women who opted for IBS (*M*_*RRM*_ = 9.1%, *SD* = 12.8 and *M*_*IBS*_ = 28.2%, *SD* = 24.8, *p* < .001; missing values: 9) with Cohen’s *d* = 0.89 (95% CI: 0.43–1.34).

#### Decisional conflict

Table 4 displays an overview on the DCS total and subscales. Both groups, RRM and IBS, displayed rather low DCS total scores, indicating low decisional conflict 6 weeks after genetic test result disclosure (T1). However, when analyzing the subgroups by choice of preventative options at T2, the RRM group showed significantly lower levels of decisional conflict at T1 than the IBS group (Table 4, *M*_RRM_ = 11.1, *M*_IBS_ = 19.8; *p* < .05) with medium effect size (Cohen’s *d* = 0.515, 95% CI: 0.01–1.02).

**Table 4:** Descriptive data and mean value comparison of DCS and its subscales by uptake of preventative measure (IBS vs. RRM) at T2

	N	*M*	*SD*	*SEM*	*p*-value
DCS Total Score					
IBS	52	19.8	17.9	2.5	.047
RRM	22	11.1	14.4	3.1	
Uncertainty					
IBS	61	33.5	29.8	3.8	.073
RRM	29	21.8	25.1	4.7	
Informed					
IBS	61	19.5	18.7	2.4	.005
RRM	30	8.3	14.5	2.7	
Values Clarity					
IBS	60	21.3	20.8	2.7	.159
RRM	28	14.6	19.7	3.7	
Support					
IBS	59	17.2	18.0	2.3	.237
RRM	28	12.5	15.8	3.0	
Effective Decision					
IBS	54	17.5	21.1	2.9	.108
RRM	26	9.9	16.1	3.2	

*Abbreviations*: *IBS* Intensified breast surveillance, *RRM* Risk reducing mastectomy, *M* Mean, *SD* Standard deviance, *SEM* Standard error of the mean, *DCS* Decisional Conflict Scale

The DCS subscales revealed that women who underwent RRM at T2 had felt more informed at T1 than women who opted for IBS (*M*_IBS_ = 19.54 vs. *M*_RRM_ = 8.33, *p* < .005). All other subscales revealed no significant differences between groups. Though not statistically significant, it is worth mentioning that the uncertainty subscale displayed higher scores in both groups compared to the other subscales, showing a tendency towards higher uncertainty among the IBS group (*M*_RRM_ = 21.8 vs. *M*_IBS_ = 33.5; *p* = .073).

#### Personality factors (FPI-R) and coping styles (FMBS)

Table 5 displays an overview on FPI-R and FMBS and their subscales. All mean values were equivalent to those of the German female population [38]. Only the FPI-R subscale ‘health concerns’ was associated with the decision for either RRM or IBS at T2. They were higher in the RRM group compared to the IBS group (*M*_RRM_ = 6.7 vs. *M*_IBS_ = 5.5, *p* < .05), with rather low scores in both groups [35]. Effect size was low with Cohen’s *d* = −0.447 (95% CI: -0.78 - -0.11). We found no association between FMBS coping styles (i.e., monitoring or blunting) and choice of preventative option at T2.

**Table 5:** Descriptive data and mean value comparison of FPI-R and FBMS by uptake of preventative measure (IBS vs. RRM) at T2

FPI-R	**IBS** *N* = 56			**RRM** *N* = 29			
	*M*	*SD*	*SEM*	*M*	*SD*	*SEM*	*p*-value
FPI-R 1: Life satisfaction	8.09	3.27	0.44	8.45	2.87	0.53	.618
FPI-R 2: Social Orientation	7.79	1.92	0.26	8.00	1.96	0.36	.630
FPI-R 3: Achievement orientation	7.21	2.38	0.32	7.48	2.71	0.50	.639
FPI-R 4: Inhibitedness	5.46	3.21	0.43	5.07	3.13	0.58	.588
FPI-R 5: Excitability	5.64	2.53	0.34	6.55	2.84	0.53	.135
FPI-R 6: Aggressiveness	2.93	2.21	0.29	3.03	2.23	0.41	.835
FPI-R 7: Strain	5.75	3.51	0.47	5.69	3.87	0.72	.942
FPI-R 8: Somatic complaints	3.96	2.80	0.37	3.48	2.72	0.51	.450
FPI-R 9: Health concern	5.54	2.42	0.32	6.66	2.38	0.44	.045
FPI-R 10: Frankness	6.25	2.49	0.33	5.72	2.55	0.47	.362
FPI-R E: Extraversion	6.59	3.28	0.44	7.38	2.80	0.52	.272
FPI-R N: Emotionality	5.54	3.61	0.48	5.48	3.37	0.63	.948
FMBS	**IBS** *N* = 51			**RRM** *N* = 28			
	*M*	*SD*	*SEM*	*M*	*SD*	*SEM*	*p*-value
Controllable Monitoring	11.92	1.91	0.27	12.32	2.09	0.40	.392
Controllable Blunting	2.84	2.10	0.29	2.75	1.60	0.30	.839
Uncontrollable Monitoring	10.90	2.81	0.39	11.11	2.91	0.55	.760
Uncontrollable Blunting	5.55	2.61	0.37	6.04	3.08	0.58	.460

*Abbreviations*: *IBS* Intensified breast surveillance, *RRM* Risk reducing mastectomy, *M* Mean, *SD* Standard deviation, *SEM* Standard error of the mean, *FPI-R* Freiburg Personality Inventory, *FMBS* Frankfurt Monitoring and Blunting Scale

#### Predictors for the uptake of preventative measures

To further investigate influencing factors for the uptake of preventative options, we fitted a binary logistic regression model (Table 6) using a forward stepwise approach (Wald). Factors where associations with the uptake of RRM had been indicated (*p* < .10) were included: the FPI-R-subscale *health concerns* and HADS-Anxiety mean values at T0 and T1, as well as the DCS total score and its subscales *informed* and *uncertainty*, the latter being not statistically significant with *p* = 0.07. In addition, we included educational level (*p* = .083).

**Table 6:** Binary logistic regression model forward stepwise approach

Step		B	S.E.	Wald	*df*	Sig.	Exp(B)	95% CI		−2 Log likelihood	Cox & Snell *R*^2^	Nagelkerke *R*^2^
								Lower	Upper			
0	Constant	−0.817	0.275	9	1	0.003	0					
1	Baseline Anxiety	0.226	0.083	7.447	1	0.006	1.254	1.066	1.476	67.638	0.132	0.186
	Constant	−2.349	0.658	12.731	1	<.001	0.095					
2	Health Concerns	0.315	0.132	5.710	1	0.017	1.371	1.058	1.776	60.970	0.221	0.311
	Baseline Anxiety	0.239	0.089	7.233	1	0.007	1.27	1.067	1.512			
	Constant	−4.388	1.164	14.207	1	<.001	0.012					
3	Health Concerns	0.388	0.149	6.827	1	0.009	1.475	1.102	1.973	54.482	0.298	0.421
	Baseline Anxiety	0.278	0.096	8.487	1	0.004	1.321	1.095	1.593			
	Decisional Conflict	−0.063	0.029	4.857	1	0.028	0.939	0.887	0.993			
	Constant	−4.296	1.229	12.216	1	<.001	0.014					

An overview is presented in Table 6. In the final model (step 3, *Χ*^2^ (*df* = 3) = 21.931, *p* < .001), anxiety at baseline, as well as decisional conflict (DCS total score) and health concerns (FPI-R) were found to be associated with the decision to undergo RRM or opt for IBS. The variance of uptake of preventive measures was explained to 42.1% (Nagelkerke *R*^2^ = 0.421, Cox & Snell *R*^2^ = 0.298) by the predictors. Women with higher anxiety scores at T0 were more likely to opt for RRM (OR = 1.321, *CI*: 1.095–1.593). Decisional conflict was negatively associated with RRM (OR = 0.939, *CI*: 0.887–0.993), indicating that higher decisional conflict is associated with opting for IBS rather than RRM. The personality trait ‘health concern’ was positively associated with the uptake of RRM (OR = 1.475, *CI*: 1.102–1.973), while educational level (*p* = .102), HADS-anxiety at T1 (*p* = .786), and the DCS subscales ‘uncertainty’ (*p* = .849), and ‘informed’ (*p* = .500) were discarded by the model.

## Discussion

This study provides insight into psychological and personality factors associated with the decision on preventative measures following *BRCA1/2* pathogenic variant detection.

Our findings suggest that higher levels of anxiety at baseline are positively associated with the uptake of RRM. This supports previous evidence [41], while Van Driel et al. had found no relation between women’s anxiety levels prior to genetic counseling uptake of RRM [28]. In addition, we found that women with a higher level of health concerns and lower levels of decisional conflict were more likely to opt for RRM. Socioeconomic status and personal BC history was not associated with the uptake of RRM, the latter confirming previous evidence that BC status (unaffected vs. affected) is not a predictor for RRM [25].

Anxiety levels in our cohort were higher than in the general population, which is in line with previous evidence [42], though not as high as reported there. This discrepancy may be caused by the high attrition rate of women with increased anxiety levels. Previous evidence has shown that psychological distress increased temporarily after genetic test result disclosure [43, 44], which was also the case in our cohort. However, in the subgroup of women opting for IBS, anxiety levels increased slightly over the course of 6–8 months after genetic test result disclosure. For the subgroup of women undergoing RRM, anxiety levels declined heavily over time, ranging near the general population’s score at T2. This is in line with previous research that demonstrated a decrease of distress levels after undergoing RRM [41]. It is of note that Julian-Reynier et al. [45] described similar trajectories with the effect of RRM or IBS on risk perception over time.

Women in our study group who underwent RRM at T2 had shown lower decisional conflict at T1 than women opting for IBS. This is reasonable, because the decision to perform prophylactic surgery is irreversible, so once the decision to undergo RRM is made, decisional conflict regarding preventative options is no longer an issue for most women. In contrast, women opting for surveillance may see their choice as a transitional solution decision (“for now”) where RRM may still be an option in the future, explaining the elevated level of decisional conflict in those women. When considering the subscales separately, the RRM group reported feeling more certain and informed about their choice compared to the IBS group. This indicates that women who opted for IBS may benefit from additional information material and counseling.

Contrasting previous evidence [17, 24, 46], our data showed no significant association between the choice of a preventative option and a close relative’s BC disease or demise. While the effect of coping styles on cancer related information needs [30] and cancer worry [31] has been reported, in our cohort of *BRCA1/2* PV carriers, coping styles were not associated with decision-making on preventative options.

### Study limitations

This study is subject to limitations. Firstly, we observed a high attrition rate from T0 through T2 and incomplete questionnaires. A total of 61 out of 159 women (38.4%) did not return the questionnaires at all three time points. This may be attributed to the emotional strain put on women receiving a positive *BRCA1/2* test result, it being an exceptional situation requiring women to deal with highly complex information and decisions, at times even under time constraints (esp. in the context of reproductive and/or therapeutic decisions). This, in addition to some women undergoing therapy for BC, may result in limited resources for additional efforts (such as participating in a study), possibly explaining why a high number of women were not able to participate at all measurement time points. Our presumption is supported by the non-responder analysis of T0 data (before genetic test result disclosure), showing significantly higher levels of anxiety in women non-respondent at every time point. When interpreting the results, it should be taken into account that non-responders were on average more anxious than women who participated in the study, which may especially limit our findings on decreased anxiety levels after RRM. As an additional consequence of the high attrition rate, our sample is smaller than targeted, resulting in low cell sizes and compromised power, a common problem with studies in the *BRCA1/2* context [17], especially for the analysis of subgroups. In addition, we employed multiple testing which may be responsible for some statistically significant results.

A second limitation is the rather short follow-up period of 6–8 months. The time lapse between genetic test result disclosure and the decision to undergo prophylactic surgery may span from a few months to several years [47]. Especially for the subgroup of (young) BC-unaffected women, a 6–8-month follow-up may not be an adequate time span for these women to reflect on the process of decision-making regarding prophylactic surgery. A longer follow-up period of several years is necessary, and should be accompanied by measurements on patient reported outcomes post-surgery (e.g., using the post-surgery questionnaire BREAST-Q). In addition, time has been reported to play a significant role for post-testing distress levels [48]. Hence, we can only provide limited evidence on the question of when the actual decision on RRM is being made.

Five women reported on current mental health conditions. No psychodiagnostic interview was performed to confirm the self-reported diagnoses. These women were not excluded from the analysis, potentially biasing our results towards elevated anxiety levels. Lastly, we did not discriminate between women from families with a known *BRCA1/2* PV and women who were the first in their family to be tested for a PV. Anxiety levels at baseline (T0) may correlate with the perceived likelihood of carrying a PV, which may differ in women from families with PVs in *BRCA1/2*. In addition, type of pathogenic variant (*BRCA1* or *BRCA2*) may play a role in decision making [49], as BC lifetime risks are higher for *BRCA1* PV carriers [1] - a factor not considered in our analyses.

The use of the HADS to measure anxiety/distress in patients with somatic and psychogenic illnesses is common and generally considered adequate for screening [50]. However, more specific tools may be used complementarily to address further aspects of psychological distress, e.g., the Cancer Worry Scale (CWS) for cancer specific distress, or the Impact of Event Scale (IES) to assess the impact of traumatic events [51].

### Clinical implications

Anxiety is an important component for clinicians to consider when counselling women with *BRCA1/2* pathogenic variants on preventative measures for BC. While elevated levels of anxiety may lead women to prefer RRM, this may not be an irrational choice. In our study, anxiety levels decreased significantly after undergoing RRM, which is reasonable considering the heavy decrease in risk of developing BC [8, 9, 52]. However, RRM is an irreversible surgical intervention and a number of serious adverse effects have been reported by women undergoing RRM [16, 21, 22]. Even though most women do not express regret relating to their decision to undergo prophylactic mastectomy, and quality of life post-surgery is high [53], women report on adverse effects caused by RRM that compromise important aspects of life [17, 53]. In the process of decision-making, personal preferences, and expectations should be taken into account and discussed, along with risks and benefits relating to risk management options.

In addition, Glassey et al. [17] reported on information gaps and the need to provide more information during the process of decision making on preventative measures. Hence, it might be beneficial to assess anxiety during the process of decision-making, as increased levels of anxiety might hamper the reception of clinical information. In addition, providing medical information is a challenge, as only a few pieces of information can be absorbed per conversation [54] – a challenging contrast to the wealth of information available in the context of hereditary breast and ovarian cancer. Patients should be guided through the process of decision making by providing written and spoken information not only on medical facts regarding RRM and IBS, but also psychological insights. In addition, training courses that enable clinicians to adapt different communication strategies may help reducing anxiety and uncertainty in counselees.

Our results indicate that women with PVs in *BRCA1/2* opting for IBS may still be in the process of decision-making and may have postponed their decision on prophylactic measures to a later point. Clinicians should be sensitized to the potential rise of anxiety levels in women with PVs in *BRCA1/2* opting for IBS, and assess and meet information and counselling needs during annual IBS appointments. For instance, increased anxiety levels may at least partly be caused by false positive results, which are not negligible [6]. Therefore, future studies should address the importance and effect of comprehensive information about the significance of such findings. Considering anxiety levels, health concerns and personality factors enables clinicians to be more responsive to women’s emotional state and to offer counseling that is more individual. High persistent anxiety levels in women carrying PVs in *BRCA1/2* should be taken into account when developing concepts for counseling these women. To identify women that may profit from additional psychological or medical counseling, respectively, psychological screenings may be carried out before and after preventative measures.

## Data Availability

Research data will be available to researchers upon reasonable request.

## Abbreviations

BC: Breast cancer
BOADICEA: Breast and Ovarian Analysis of Disease Incidence and Carrier Estimation Algorithm
BRRM: Bilateral risk-reducing mastectomy
CI: Confidence Interval
CRRM: Contralateral risk-reducing mastectomy
CWS: Cancer Worry Scale
DCS: Decisional Conflict Scale
FMBS: Frankfurt Monitoring and Blunting Scale
FPI-R: Freiburg Personality Inventory
HADS: Hospital Anxiety and Depression Scale
HADS-D: German version of the Hospital Anxiety and Depression Scale
IBS: Intensified breast surveillance
IES: Impact of Event Scale
M: Mean value
MBSS: Miller Behavioral Style Scale
OC: Ovarian cancer
OR: Odds Ratio
PV: Pathogenic germline variant
RRM: Risk-reducing mastectomy
SD: Standard deviance
SEM: Standard error of the mean
